# Green Buildings and Health

**DOI:** 10.1007/s40572-015-0063-y

**Published:** 2015-07-10

**Authors:** Joseph G. Allen, Piers MacNaughton, Jose Guillermo Cedeno Laurent, Skye S. Flanigan, Erika Sita Eitland, John D. Spengler

**Affiliations:** Harvard T.H. Chan School of Public Health, 401 Park Drive, 404-L, Boston, MA 02218 USA

**Keywords:** Green buildings, Indoor environmental quality, Health, Systematic review

## Abstract

Green building design is becoming broadly adopted, with one green building standard reporting over 3.5 billion square feet certified to date. By definition, green buildings focus on minimizing impacts to the environment through reductions in energy usage, water usage, and minimizing environmental disturbances from the building site. Also by definition, but perhaps less widely recognized, green buildings aim to improve human health through design of healthy indoor environments. The benefits related to reduced energy and water consumption are well-documented, but the potential human health benefits of green buildings are only recently being investigated. The objective of our review was to examine the state of evidence on green building design as it specifically relates to indoor environmental quality and human health. Overall, the initial scientific evidence indicates better indoor environmental quality in green buildings versus non-green buildings, with direct benefits to human health for occupants of those buildings. A limitation of much of the research to date is the reliance on indirect, lagging and subjective measures of health. To address this, we propose a framework for identifying direct, objective and leading “Health Performance Indicators” for use in future studies of buildings and health.

## Introduction

### Background

It is well-known and oft-repeated in environmental health circles that we spend 90 % of time indoors [1–3]. Because this constitutes the vast majority of our exposure time, and concentrations of many indoor pollutants are actually higher indoors than outdoors, it follows logically that indoor environments influence our health [3]. Over 40 years of research on the indoor environment has yielded many insights into building-related factors that influence health, well-being, and productivity [4, 5]. This manuscript is not intended to include a review of these factors in detail, but we cite a few important aspects here to highlight the breadth of the issue: environmental hazards (radiological, chemical, biological, physical) [6], building design, (ventilation, pressurization, filtration, lighting, acoustics) [6, 7], social factors (location, safety) [8], behavioral factors (curriculum, work activities, wellness programs) [9], adjacent land use (chemical releases, walkability, noise sources, green spaces) [10, 11], architectural design (physical activity promotion, eating spaces, material selection, biophilic design and access to natural lighting) [12, 13], and operations and maintenance (preventative maintenance, upkeep, cleaning, integrated pest management) [14, 15, 16••]. Environmental health research tends to focus on the potential for adverse health effects from indoor exposures—for example, radon and lung cancer [17], phthalates and asthma [18, 19], second-hand smoke and increased risk of premature death [20]. Discussed less frequently is the corollary—the fact that the indoor environment can provide health benefits when we optimize building environments for human health [21].

The green building movement was born out of this recognition that buildings have the potential for both positive and negative impacts on people and the environment, and the desire to mitigate negative impacts while enhancing those features that provide positive benefits [22]. Green buildings minimize environmental impacts largely through energy and water conservation measures, and through limiting local impacts to the building site. However, green building design also focuses on improving human health. Green buildings influence human health at two critically important scales: directly at the individual level through providing optimized indoor environments, and indirectly on a population level through reductions in energy use and thus reductions in air pollutants that cause premature death [23, 24], cardiovascular disease [25, 26], exacerbate asthma conditions [27] and contribute to global climate change, itself associated with a cascade of adverse human health impacts [28••, 29].

### Green Building Benchmarks

The green building revolution is currently underway, with the leading green building certification reporting more than 3.6 billion square feet certified to date [30]. That certification, Leadership in Energy and Environment Design (LEED®), was started in August of 1998, and, since that time, there are more than 69,000 LEED building projects in over 150 countries [30]. An in-depth review and discussion of LEED is beyond the scope of this paper. However, despite the multiple rating systems, there are commonalities in the credits that are related to health across the rating system. Most of the LEED credits that are directly linked to individual occupant health fall under the Indoor Environmental Quality category. Required are ventilation rates that meet ASHRAE 62.1 (“Ventilation Rates for Acceptable Indoor Air Quality”; [31]), control of environmental tobacco smoke and cleaning practices to limit biological and chemical exposure. There are also health-related credits available for enhanced IAQ strategies (e.g., increased filtration, CO_2_ monitoring), increasing ventilation, use of low emitting materials (e.g., low-VOC paints and furnishings), protecting ventilation systems from construction debris, indoor air quality monitoring (e.g., formaldehyde, particles, VOCs), thermal comfort, interior lighting, daylighting and views, integrated pest management, and green cleaning [32].

In addition to LEED, there have been other recent efforts to develop building standards that focus on health. For example, the WELL Building Standard, released in 2014, was developed over a 7-year period by the International WELL Building Institute, and focuses predominately on occupant health; there are no credits for energy or water conservation [33]. The standard specifically takes a biological systems approach and incorporates the following components of health—air, water, nourishment, light, fitness, comfort, and mind. Similar to LEED, there are credits for ventilation, air quality, lighting, acoustics, and thermal comfort. WELL also includes requirements for carbon filters (air and water), drinking water quality, sleep quality and ergonomic factors, among others. WELL also extends the chemical focus to include environmentally persistent organohalogen and semi-volatile compounds, limiting the use of halogenated flame retardants, polyfluorinated chemicals, and phthalates. The Living Building Challenge (LBC) is another health-focused certification program, created by the International Living Future Institute in 2006 [34]. Similar to the other two standards, the framework of this certification program is broken down into a number of categories, or “petals.” The Living Building Challenge 3.0 consists of the following petals: place, water, energy, health and happiness, materials, equity, and beauty. An important contribution to the green building movement is the LBC’s establishment of the “Red List,” which follows the precautionary principle on banning the use of harmful materials or chemicals, and the “DECLARE” process, which requires disclosure of ingredients in products.

The benefits of green building on energy and water conservation are well researched and recognized [35•]. However, the indoor environmental quality and human health benefits of green buildings have not been as thoroughly evaluated. Therefore, our objective was to examine the current state of evidence regarding green buildings and health. First, we reviewed the scientific literature for research that specifically examines green buildings, indoor environmental quality, and health. Second, we propose a framework and metrics for evaluating health in buildings.

## Review of Existing Literature on Green Buildings and Health

### Methodology

We conducted a search for research studies that specifically focused on exploring relationships between green buildings and health. Inclusion criteria included the following: (1) peer-reviewed paper or government report, (2) evaluation of green buildings, (3) data on indoor environmental quality perception or measurements, and (4) data on health, comfort, productivity, or well-being. Our initial searches were performed using Web of Science and PubMed, with keywords that included the following: green buildings, LEED, WELL, Living Building Challenge, high performance buildings, indoor air quality, indoor environmental quality, and health. The next step in our process was to review the reference sections of the papers identified from the first search and select additional relevant studies. This was repeated again with this second round of studies. Finally, we conducted internet searches for “grey” literature (e.g., government reports) that included original research studies that fit our inclusion criteria. As a result of this search process, we identified 17 studies on green buildings and health for our review. A minimum of three co-authors reviewed each study. We organized our review based on strength of the study design, starting with studies that relied solely on occupant surveys and concluding with studies that included objective measures of exposure and outcomes (Table 1). As the literature currently lacks a study where occupants are blinded to their exposure group (i.e., being in a green environment or a conventional environment), studies with self-reported metrics were deemed weaker than those with objective measures. Due to the widespread adoption of LEED and the fact that it was started 15 years ago, nearly all of the studies of green buildings we identified were focused on LEED buildings. As research is generated on other health-focused certifications and standards (e.g., WELL and LBC), an additional review paper is warranted.

**Table 1:** Overview of published studies on the relationship between green buildings and health

Study	Sample size (no. of people)	Building type(s)	Results (compared to conventional)
Studies with only occupant surveys			
Huizenga et al. [37]	Not provided	Offices	↑ Air quality
		- 3 green	↑ Cleanliness
		- 45 conventional	↑ Thermal comfort
Abbaszadeh et al. [39]	33,285	Offices	↑ Air quality
		- 21 green	↑ Cleanliness
		- 160 conventional	↑ Thermal comfort
Lee and Kim [40]	40,488	Offices	↑ Air quality
		- 15 green	↑ Cleanliness
		- 200 conventional	↑ Thermal comfort
			↓ Lighting
			↓ Acoustics
Altomonte and Schiavon [41]	21,477	Offices	↑ Air quality
		- 65 green	↑ Cleanliness
		- 79 conventional	↓ Lighting
			↓ Acoustics
Paul and Taylor [42]	93	University	↓ Thermal comfort
		- 1 green	
		- 2 conventional	
Hedge et al. [43]	319	University	↑ Ventilation
		- 2 green	↑ Air quality
		- 1 conventional	
Thatcher and Milner [44]	441	Offices	↑ Perceived ventilation
		- 1 green	↑ Air movement
		- 2 conventional	↓ Thermal comfort
			↓ Lighting
U.S. General Services Admin. (GSA) [35•]	Not Provided	Offices	↑ Occupant satisfaction
		- 22 green	↑ Thermal comfort
			↓ Acoustics
Singh et al. [45]	263	Offices	↑ Self-reported well-being
		- 2 green	↓ Absenteeism
		- 1 conventional	↓ Asthma and allergy symptoms
Studies with IEQ Measurements + Occupant Surveys			
Liang et al. [46]	233	Offices	↑ Thermal comfort
		- 3 green	↑ Air quality
		- 2 conventional	↑ Lighting
			↑ Acoustics
Ravindu et al. [47]	70	Factories	↓ Thermal comfort
		- 1 green	↓ Ventilation
		- 1 conventional	
Newsham et al. [48]	2545	Offices	↑ Air quality
		- 12 green	↓ Acoustics
		- 12 conventional	
Jacobs et al. [49•]	58	Public housing	↑ Self-reported well-being
		- 1 green rehabilitation	↑ Cleanliness
			↓ Allergens
Garland et al. [50]	Not Provided	Public housing	↓ Asthma and allergy symptoms
		- 1 green rehabilitation	
Breysse et al. [51•]	41	Public housing	↓ Respiratory symptoms
		- 1 green rehabilitation	
Colton et al. [16••]	24	Public housing	↑ Air quality
		- 1 green rehabilitation	↑ Ventilation
		- 1 conventional	↓ Asthma symptoms
Studies with Objective Health Outcome Measures			
Thiel et al. [52••]	Not Provided	Hospitals	↑ Employee satisfaction
		- 1 green	↑ Quality of care
		- 1 conventional	↓ Length of open positions
			↓ Patient mortality

### Studies with Only Occupant Surveys

The simplest way to obtain information on both indoor environmental quality (IEQ) and occupant satisfaction is through surveys. While a strength of surveys is that they can be deployed in large cohorts, a limitation is that they are subjective measures of exposure and outcomes and therefore prone to information bias (e.g., misclassification), selection bias (e.g., self-selection bias), and dependent errors [36].

Several studies of green buildings have used an occupant survey tool created by the Center for the Built Environment at the University of California Berkeley. They conducted initial pre-testing and validation of core questions through a method called “cognitive interviewing” where they assessed respondents’ comprehension of questions and accuracy of answers [37]. This method gave the CBE survey a relatively high level of validity. Over the course of 10 years, 52,980 individual occupant responses were collected in 350 office buildings (49 self-reported as LEED certified) [38].

We identified four studies that used this database to test occupant satisfaction with various IEQ parameters in LEED and non-LEED buildings [37, 39–41]. All studies found that occupants were more satisfied with indoor air quality, building cleanliness/maintenance, and their workspace in LEED buildings than in non-LEED buildings. The studies were discordant on the effects of other parameters. Huizenga et al., Lee et al., and Abbaszadeh et al. found occupants more satisfied with thermal comfort in LEED buildings while Altomonte et al. did not. Huizenga et al. and Abbaszadeh et al. found no statistically significant differences between building types for lighting and acoustics while Altomonte et al. and Lee et al. concluded that non-LEED buildings performed better. The discrepancies in results are primarily driven by the building inclusion and exclusion criteria used in each study. Each researcher analyzed a different subset of the CBE dataset based on when the study was conducted, what the required response rate was, how the responses were weighted, and which buildings were classified as LEED and non-LEED. For example, Altomonte et al. excluded non-LEED buildings built or renovated before 1998 since they predated the LEED certification system and weighted responses based on the number of responses obtained at each building, although they did not account for the correlated nature of the responses in their analysis.

Using a different survey tool, Paul and Taylor performed a survey of occupants of one green office building and two conventional office buildings on a university campus [42]. The survey measured self-report comfort and satisfaction perception. A critical difference between the green and conventional buildings that could not be controlled for in a study this small is the type of ventilation (natural vs mechanical, respectively). The authors conclude that there were no differences between the buildings, except for occupants in the green building were more likely to report being warm and more likely to describe the work environment as poor; however, detached from the conclusion is an observation that the hydronic cooling system was not working properly in the green building at the time of the study. Therefore, the conclusions regarding thermal comfort were almost certainly a result of a malfunctioning cooling system and not related to the comparison of green versus conventional buildings.

A similar study investigated the same IEQ parameters in a different set of university buildings [43]. Three hundred nine total occupants from two green (LEED Silver) and one conventional building completed surveys on their work environment and health. More occupants reported that the air was fresh and that the air quality was “good” in the green buildings compared to the conventional building, and there were with statistically significant fewer reports of coughs/sneezes and neck/shoulder ache. The occupant surveys in the two green buildings were inconsistent for most environmental conditions investigated, including air temperature, air movement, and noise. Satisfaction with ventilation, air quality, and lighting was significantly higher in the green buildings. The authors concluded that aspects of green building design can result in better perceived IEQ as rated by building occupants. They did not find evidence that the green buildings were more comfortable or productive workplaces.

Thatcher et al. collected self-reported occupant responses related to health and well-being in two groups of employees over the course of 1 year: one which crossed over from conventional offices to a Green-star accredited office after the baseline series of questionnaires and one which worked in conventional offices the entire time [44]. The authors report significantly increased physical well-being and satisfaction with many IEQ parameters for employees in the green building compared to the conventional building. Occupants of the green housing reported better perceptions of ventilation, air movement, and reductions in humidity and stale air. Lighting conditions were perceived as dimmer in the green housing.

The US General Services Administration (GSA) operates the Office of Federal High Performance Buildings [35•]. GSA conducted a survey of 22 sustainably designed buildings from representative areas of the country in order to compare performance against national averages (16 met or exceeded LEED, and 6 met Energy Star or California Title 24 Energy Standard). The survey included information on occupant experience. GSA found 27 % higher occupant satisfaction in the 22 sustainably designed buildings compared to the national average, and reported that the top third of these buildings had even greater margins (78 % higher than average). Higher satisfaction was reported for air quality, general building satisfaction, cleanliness and thermal comfort. There were no differences in satisfaction with lighting, and they found lower scores for sound privacy, but not noise level.

Singh et al. followed employees who transitioned from conventional to two LEED-certified green buildings [45]. A pre- and post-move survey was administered (two case studies; *n* = 56 and *n* = 207). Unlike the studies described previously, the questions focused on absenteeism and productivity rather than environmental perceptions. They compared results using paired *t*-tests and found that, after moving into green buildings, employees reported significantly (*p* < 0.05) lower absenteeism attributable to asthma and respiratory allergies, fewer work hours affected by asthma and respiratory allergies, fewer work hours affected by depression and stress, and increased productivity related to improved IEQ. Combining these impacts together resulted in 42.75 more work hours per year per occupant (greater than one whole work week) in the LEED building compared to the conventional building.

### Studies with IEQ Measurements + Occupant Surveys

To reduce misclassification, dependent error, and bias, subjective measures can be augmented or replaced by objective measures of IEQ and occupant well-being. Several studies have complemented occupant satisfaction with the built environment with objective measures of IEQ. Liang et al. combined a variant of the CBE survey with IEQ measurements in three green buildings in Taiwan (EEWH-certified) to two conventional buildings [46]. They found improvements in thermal conditions, indoor air quality, noise, and lighting, all of which contributed to higher occupant satisfaction scores. Ravindu et al. conducted a case study of self-reported survey results in a LEED-certified factory and a second factory used as a control in Sri Lanka [47]. They report thermal quality and ventilation as less satisfactory in the green factory and no difference in acoustics or air quality. The survey was performed on 35 workers randomly selected from each facility, but then matched based on work location and type of work. Matching was not performed for age or other potential confounding variables (gender, years worked, supervisor). Newsham et al. matched 12 green buildings to 12 comparable conventional buildings [48]. At each building, an IEQ assessment was conducted and occupants completed a questionnaire about their well-being. The green buildings performed slightly worse for noise and better for indoor air quality, with other IEQ parameters being largely consistent. Occupants in green buildings reported higher satisfaction with access to outside views, better mood, better sleep quality at night, and fewer visual or physical discomfort reports.

Several studies in public housing have used self-reported health metrics in conjunction with occupant satisfaction with IEQ to better characterize well-being. Jacobs et al. combined the use of validated surveys of physical and mental health with objective measurements of allergens [49•]. Measurements were taken at baseline and 1 year after study subjects moved to a renovated space certified LEED Gold. Cockroach allergen (Bla g1) and mouse allergen (Mus m1) registered significant sustained reductions 3 months after the intervention. The 58 participants who participated in both measurements reported an overall improvement in their health of 8 %. Another study followed tenants in an affordable housing for 18 months after moving into a LEED-certified complex [50]. The extent of the renovation included the substitution of gas stoves for electric units, integrated pest management as well as the use of biodegradable cleaners; no smoking is permitted in the complex premises. The main finding from this study was a significant decrease in daytime respiratory symptoms and nighttime asthma symptoms after moving into the LEED-Platinum certified complex.

Breysse et al. used validated surveys from the Centers for Disease Control and Prevention and the National Institute of Environmental Health Sciences to study the effectiveness of green renovations in public housing [51•]. Significant decreasing trends in the number of reported non-asthma respiratory problems (e.g., emphysema, hay fever, sinusitis, and chronic bronchitis) prevailed for 18 months after moving into the renovated space, both in children and adults. The overall health status of adults and children also improved, although the effect was only significant for the adults. The authors suggest that the improvement in respiratory health outcomes was not stronger presumably because ventilation levels were lower than expected (982 ppm CO_2_ mean annual concentration) in the green housing. One considerable limitation of this study is that pre-renovation information relies on the participants’ recall once they had already moved to their renovated unit.

Colton et al. investigated the difference in IEQ parameters and self-reported health between tenants of conventional housing and affordable green housing [16••]. The study also included a subset of residents transitioned from conventional to green housing. Although thermal comfort was perceived less satisfactory and air changes per hour were lower in the green homes, there was a 47 % reduction in the reported sick building syndrome symptoms among green housing tenants [16••]. Environmental sampling also showed significantly lower PM_2.5_, NO_2_, and nicotine in green homes compared to conventional apartments, despite AER being lower in the green homes. CO_2_ concentrations in green units (median = 1204 ppm) were considerably higher than benchmark values of adequate ventilation. Other benefits include lower reports of pests, fewer water-related issues, and fewer inadequate ventilation issues [16••].

### Studies with Objective Health Outcome Measures

Objective health metrics are even more important than objective measurements of IEQ because the relationship between building design and IEQ is currently better understood than the relationship between building design and health. Thiel et al. compared a newly constructed LEED-certified hospital to a conventional hospital in Pittsburgh, PA [52••]. Because of their unique focus on hospitals, they had the most objective health assessment of the studies reviewed; hospital records on both the patients and employees were compared at the two hospitals. A key strength of this study is that the authors had 10 and 3 years of objective, standardized hospital metrics in the conventional and green hospital, respectively. The green hospital had a 19 % decrease in mortality despite an 11 % predicted increase based on the severity of cases drawn to the new facility. Employees were generally more satisfied with the newer facility based on an increase in employee tenure, a decrease in employee turnover, and a decrease in the length of open staff positions. The quality of care also improved in the new facility: blood stream infection rates declined 70 % and number of corrections to the Medication Administration Record declined 49 %. The authors conclude that the results, “lead to a reasonable observation that the facility did in fact contribute to the overall improvements.” Without measurements of IEQ in this study, it is not possible to identify the specific green building attributes that were responsible for the improvements.

## Health Performance Indicators

The studies of green buildings conducted to date, reviewed in this manuscript, were all attempting to answer a seemingly straightforward question—are green buildings healthier buildings? A related, more generalized set of questions are simply—what constitutes a healthy building, and how do we measure this? Determining what metric or metrics best capture the health of an occupant in a building is an important challenge that we as health researchers all face. Many of the studies in our review asked occupants to self-report on their health and perceptions of the indoor environment. Several measured indicators of indoor environmental quality performance as an indicator of health (e.g., ventilation, VOCs, particles), and a few used true, objective measures of occupant health (e.g., standardized healthcare performance metrics). Here, we propose a framework for conceptualizing these and other metrics for studying health in buildings, borrowing a business term: key performance indicator (KPI).

**Fig. 1:**
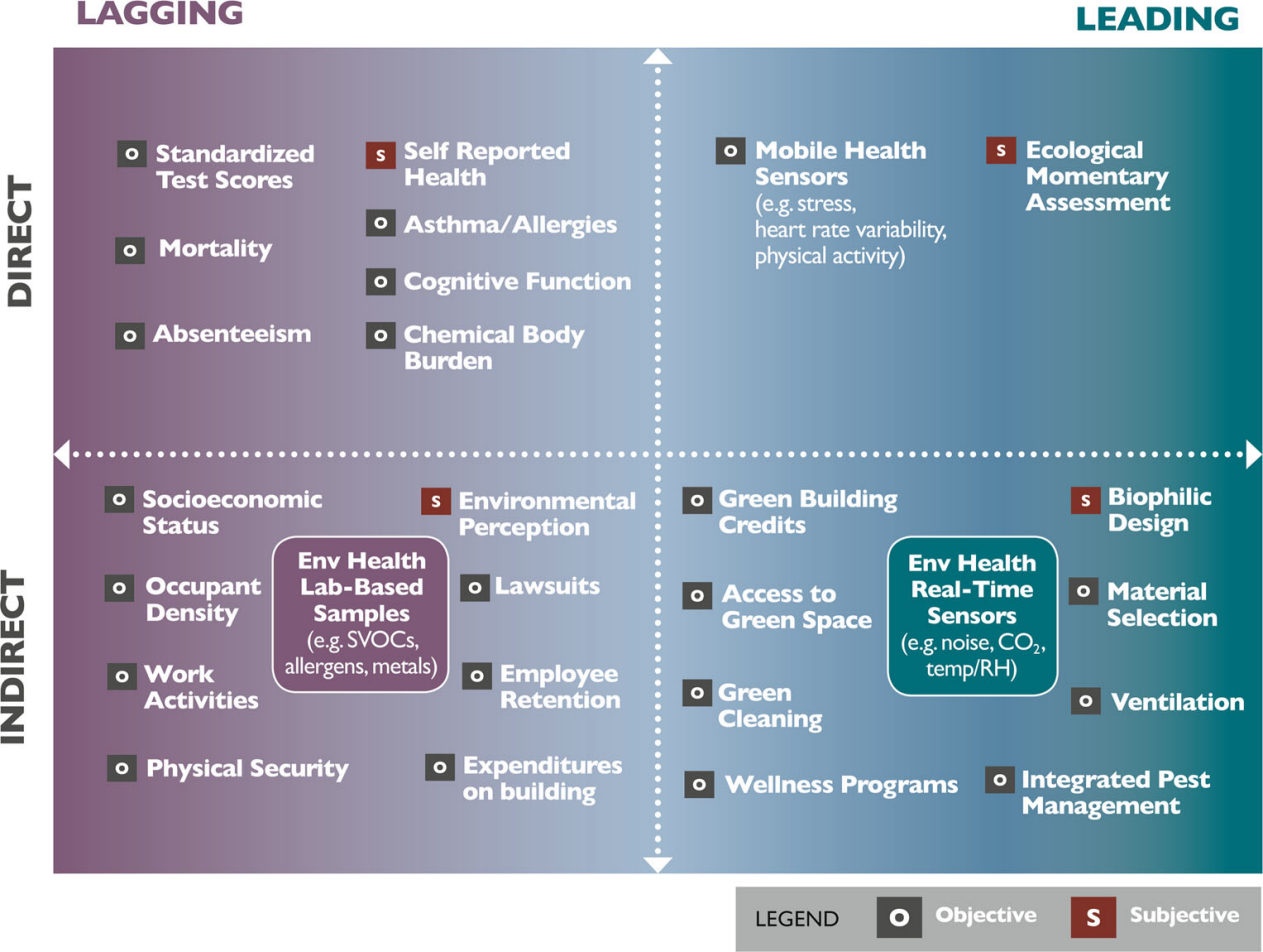
Health Performance Indicators framework with example metrics

In a recent article in the Harvard Business Review, Maubossin defines KPIs as metrics that companies use to quantify, communicate, and ultimately manage business performance [53]. The goal is to create value, but Maubossin’s research highlights some common pitfalls in choosing metrics, including failing to rely on objective data (what he describes as intuition-based decision-making, or “overconfidence”), and using metrics simply because we can measure them (what he calls “availability”) [53]. The same concepts and potential problems apply to measuring health in buildings. Here, we re-brand “KPI” and propose the use of the term “Health Performance Indicators” (HPIs). HPIs are the quantifiable measures of human health that can be used to identify drivers of negative and positive impacts of buildings on health, productivity and well-being of occupants. We further propose that HPIs be divided into those that are direct versus indirect measures of health, those that are objective and subjective measures, and those that are leading versus lagging indicators of health, well-being, and productivity in buildings (Fig. 1). The goal is to be explicit about what we are measuring, why we are measuring it, and how this information helps us understand, and ultimately improve, health of people in buildings.

We believe that a common set of HPIs apply to nearly all buildings. For example, building design and performance (e.g., green building, ventilation), measures of environmental health (e.g., chemicals, biologicals, radiological hazards), and measures of occupant health (e.g., self-reported health, objective physiological measures, asthma). Our research, and the research of countless other health scientists, has informed the inclusion of many other example HPIs into this figure, from the obvious (e.g., ventilation), to the more obscure (e.g., lawsuits). For schools, the same baseline factors are important, but school-specific metrics would also likely include teacher health, student absenteeism, standardized test scores, and other school-specific factors. For hospitals, this may include patient recovery, staff performance, and infection rates. Our intent was not for this to be a definitive or exhaustive list; least important is exactly where in the quadrants each parameter falls. Rather, this framework is intended to provoke researchers to examine the health-related metrics they use, what those metrics are actually telling us about health (direct vs indirect), the strength of the metrics (objective vs subjective), and what the metrics allow us to do in terms of timely interventions (leading vs lagging). The proliferation of mobile health sensors, sometimes termed *mHealth* (mobile health) or referred to as the *Quantified Self* movement, is enhancing our ability to obtain objective, leading and direct measures of health of occupants of buildings. Last, we hope this framework stimulates health researchers to evaluate their metrics in order to avoid the “availability” and “overconfidence” issue.

## Conclusion

Overall, the initial scientific evidence published to date indicates better measured and perceived indoor environmental quality and health in green buildings versus non-green buildings. For indoor environmental quality, green buildings had lower levels of VOCs, formaldehyde, allergens, ETS, NO_2_, and PM. Many of these environmental contaminants that have been linked to adverse health effects are explicitly addressed in green building design credits, so these early findings suggest that the design elements targeted at improved IEQ translate to significant reductions in actual exposure. Building acoustics was the one IEQ parameter that did not consistently score better in green buildings; in several studies, participants reported lower satisfaction with noise.

The IEQ benefits in green buildings translate to better self-reported health outcomes across several indicators. This includes fewer sick building syndrome symptoms, fewer respiratory symptom reports in children, and better physical and mental health. Occupants also report benefits that indicate improved work productivity in green buildings. In one study, the occupants reported fewer absenteeism and fewer work hours affected by asthma and allergies in green buildings. Also related to productivity, green buildings were associated with lower employee turnover and a decrease in the length of open staff positions. In a hospital setting, they noted improved quality of care in green buildings, fewer blood stream infections, improved record keeping, and lower patient mortality.

These initial research studies on green buildings have several important limitations. Nearly all of the studies rely on self-reported and subjective measures of health and in all cases occupants know their exposure status. The lack of blinding does not bias the IEQ measurements, but could impact self-report measures, which constitute the bulk of the outcomes assessed in the literature. Also, many of these studies are case studies or have small samples sizes which increase the chance for type II error (i.e., low statistical power). Last, the lack of reporting information on the specific green building credits precludes an analysis of the design features that contribute to improved IEQ and health.

Designing for health is becoming an increasingly important part of what it means to be a green building, as evidenced by the recent inception of WELL and LBC. As the researchers begin assessing the success of these programs in relation to LEED-certified and conventional buildings, the need for high quality health metrics will become paramount. Our proposed framework for identifying HPIs in buildings, which characterizes indicators as objective or subjective, direct or indirect, and leading or lagging, is useful when designing studies that attempt to identify specific building-related attributes, in green buildings or otherwise, that lead to improved occupant health.
